# Epiphanies, velcro balls and McDonaldization: highlights from the 5^th ^Meeting of the International Society for Behavioral Nutrition and Physical Activity

**DOI:** 10.1186/1479-5868-3-30

**Published:** 2006-09-21

**Authors:** Kylie Ball

**Affiliations:** 1 President, International Society for Behavioral Nutrition & Physical Activity, Deakin University, Melbourne, Australia

## Abstract

This commentary provides an overview and selected highlights from the scientific program of the 5th Annual Meeting of the International Society for Behavioral Nutrition and Physical Activity.

How can the years' worth of international, cutting-edge behavioral nutrition and physical activity research, conceptual and methodological advances, and inspiring intellectual debate that comprised the program of the 5^th ^Annual Scientific Meeting of the International Society for Behavioral Nutrition and Physical Activity (ISBNPA), be adequately summed up in just a few short paragraphs? Clearly this is a near impossible objective, and instead I hope to convey in this commentary just a flavour of the diverse, high-calibre and motivating program featured at this recent event.

Delegates at the Meeting held in Boston on July 13–16 contributed and were treated to all of the above, and more. With six world-renowned keynote speakers; a spirited debate; 16 symposium sessions; more than 150 peer-reviewed oral papers and poster presentations; 3 cutting-edge practical workshops; and networking opportunities with over 250 delegates from 27 countries around the world on the menu, the meeting certainly lived up to the highest of expectations.

Among the conference highlights was the focus on research themes of increasing importance internationally. The International Society for Behavioral Nutrition and Physical activity was formed in recognition of the significant impact these two key behaviors have on health. While sedentary lifestyles and poor diet pose a range of health risks, currently there is a global focus particularly on those risks posed by an 'obesity pandemic', to which physical inactivity and certain dietary behaviors are arguably key contributors. This theme was reflected in two keynote sessions, from Professors Jim Hill and Steve Gortmaker, and a number of symposium and free paper sessions, discussing issues such as the behavioral causes of obesity; the potential contributing roles of individuals, parents, schools, regulators, and the broader environment; and opportunities for obesity prevention and intervention with children, adolescents and adults. A particular highlight was the inspiring session on obesity in Latin America, in which Juliana Kain, Juan Rivera and Kim Gans identified the very concerning rapid increase in obesity rates in countries such as Chile and Mexico, as well as among Hispanic adults living in the USA. The very promising results of early intervention programs to address this problem amongst school-children in Latin America at least provide some hope for the future, and the successes and challenges facing public health researchers working in these regions were inspiring to hear. The Society also prides itself on encouraging new paradigms by which to consider issues related to behavioral nutrition and physical activity, and this was evident, for example, in the session on *Health at every size: a new weight paradigm for obesity and weight-related issues*, chaired by Dr Marie-Claude Paquette, which suggested that an approach emphasizing health, acceptance of body size, physical activity and normalized eating, rather than 'weight loss', may be the antidote to the obesity epidemic.

The ISBNPA, however, is certainly not simply 'another obesity society', but draws together international expertise in investigating and intervening with nutrition and physical activity behaviors. A second key research theme illustrated in many outstanding presentations was the significant advances achieved in understanding the determinants of these behaviors. Environmental determinants, including built, natural, socio-cultural and policy factors, were a particular focus, and delegates heard from some of the world's foremost experts in the advanced approaches and methods used to investigate such determinants. These included the thoughtful keynote session given by Dr S.V. Subramanian, one of the world's leading experts in multilevel statistical methods, on the importance of social and neighborhood context and health, and the value of new methodologies (from multilevel modeling to the 'McDonaldization' scale!) for enhancing our understanding of these contextual determinants. Exciting methodological advances in measuring behavioral determinants were also provided in the challenging and thought-provoking symposium on Item-response theory, chaired by Dr Louise Masse.

For many of us working in the fields of behavioral nutrition and physical activity, our ultimate professional aims include the development of sufficient knowledge and understanding to enable us to modify health-compromising behaviors and promote nutrition and physical activity to optimum levels. Evidence of substantial gains in these pursuits was abundant at the meeting. For instance, the impressive Pro-Children study has achieved numerous successes in promoting fruit and vegetable consumption in children across Europe, as outlined in the symposium chaired by Professor Hans Brug. Important achievements in behavior change interventions in worksites (symposium chaired by Professor Simone French), general practice (chaired by Dr Torben Jorgensen) and using computer tailoring (chaired by Willemieke Kroeze) were also showcased. Nonetheless, it is clear that we still have some way to go in our efforts to effect health-promoting behavior change. For instance, the need for truly theoretically-driven behavior change interventions, in which clearly defined behavior change techniques are systematically tested, is necessary in order to shift our intervention efforts from 'inventive art' to 'experimental science', as argued eloquently in the keynote session given by Professor Charles Abraham. For many delegates, a key highlight of the meeting was the riveting keynote debate in which the value of conventional versus more novel theoretical models was politely but assertively posed and challenged by Professors Hans Brug and Ken Resnicow. A little controversy; the questioning of conventional thinking and stirring up and reviewing of established ideas are surely signs of a successful conference, and the keynote debate certainly hit the mark in this respect. Professor Resnicow's introduction to 'Chaos Theory', in which juggling Velcro balls leading to an 'epiphany' provided a useful analogy for the random, chaotic, and non-linear process by which many individuals arrive at the 'tipping point' for behavior change, was particularly novel and well-received. A paper based on Resnicow's presentation has now been published in the International Journal of Physical Activity and Nutrition [1], with commentaries by Hans Brug [2] and the chair of the keynote debate session, Tom Baranowski [3].

The conceptual innovations evident in the themes described above were complemented in the program with discussions and examples of cutting-edge methodological advances. Three pre-conference workshops provided the ideal opportunities to facilitate discussion and dissemination of practical applications of these methods. For example, the keynote on social context and health highlighted methodological advances in analytical techniques valuable for the consideration of environmental and contextual influences on health behavior, complemented by the inclusion in the program of the world-renowned workshop on multi-level statistical modelling, presented by Drs Frank van Lenthe and Jos Twisk. Similarly methodological themes described in the symposium on Applications of Item Response theory were explored in detail in the practical workshop on Measurement Concepts and Methods arising from IRT, facilitated by Drs Diane Allen and Louise Masse. Finally, the theme of childhood obesity and opportunities for parental coaching in its management was examined further in the workshop offered by Dr Moria Golan.

Certainly among the many enjoyable aspects of the ISBNPA program was the announcement of the awards to encourage student and early career members of the Society. Congratulations are due to Ellen Haug, University of Bergen, Norway, and Dr Eric Hodges, Baylor College of Medicine, USA, for best oral presentations; and to Anne-Marie Meyer, University of North Caroline at Chapel Hill, and Dr Charlotta Pisinger, Research Centre for Prevention and Health, Denmark, for best poster presentations from student and early career researchers. Students and early career researchers were also treated to a 'Meet the mentors' event, in which they had the opportunity to participate in small-group discussions on research career-related issues with five seasoned researchers in the field from around the world.

In summary the 5^th ^ISBNPA meeting was a resounding success from which delegates walked away inspired, challenged, informed, and hopefully with many new ideas, new colleagues and new friends. Thanks are due to hard-working local organizers; the ISBNPA Program and Executive Committees; the many generous meeting sponsors; and most of all to the conference delegates. The success of any scientific meeting rest heavily on the efforts of attending delegates, and I heartily thank all delegates for such valued contributions to the program and to the International Society of Behavioral Nutrition and Physical Activity.
